# Case Report: Chronic myelomonocytic leukemia with mature plasmacytoid dendritic cell proliferation initially classified as MDS-IB2

**DOI:** 10.3389/fonc.2026.1961676

**Published:** 2026-10-22

**Authors:** Junhong Chen, Juan Liu, Yanying Ren

**Affiliations:** Department of Medical Laboratory, Dazhou Integrated Traditional Chinese Medicine and Western Medicine Hospital, Dazhou Second People’s Hospital, Dazhou, China

**Keywords:** case report, chronic myelomonocytic leukemia, mature plasmacytoid dendritic cell proliferation, monocytosis, myelodysplastic neoplasm

## Abstract

**Background:**

Mature plasmacytoid dendritic cell proliferation (MPDCP) is uncommon in myeloid neoplasms and can complicate diagnosis when dysplasia, increased blasts, monocytosis and pDC expansion coexist.

**Case presentation:**

An 88-year-old man presented with anemia, thrombocytopenia, and marked monocytosis. Peripheral blood and bone marrow findings showed atypical monocytes, multilineage dysplasia, 13.5% myeloblasts, and an expanded pDC-like population. Flow cytometry identified a pDC-like population accounting for 8.60%, with CD4, bright CD123, CD303 and partial CD304 expression, and absence of CD34, CD117 and CD56. Cytogenetic and molecular testing revealed a complex karyotype with monosomy 7 and a *TP53* mutation. The patient was initially diagnosed with MDS with increased blasts-2 (MDS-IB2) with MPDCP. Retrospective review of longitudinal blood counts demonstrated persistent monocytosis that predated the initial hematologic evaluation and persisted during follow-up. Together with the marrow morphology and clonal genetic abnormalities, these findings supported reclassification as CMML-2 with MPDCP.

**Conclusion:**

This case highlights the value of longitudinal monocytosis in recognizing CMML when dysplasia, increased blasts, and MPDCP complicate the initial diagnosis, and underscores the importance of distinguishing the pDC population from myeloid blasts during disease classification.

## Highlights

- Longitudinal monocytosis, together with marrow and clonal findings, supported reclassification from MDS-IB2 to CMML-2.
- Integrated morphologic and immunophenotypic assessment was essential for distinguishing MPDCP from other pDC proliferations.
- Separating pDC-like cells, immature myeloid cells, and monocytic cells was critical for accurate blast assessment and disease classification.

## Introduction

Chronic myelomonocytic leukemia (CMML) is a myelodysplastic/myeloproliferative neoplasm characterized by persistent peripheral blood monocytosis, together with morphologic dysplasia and/or evidence of clonality (1, 2). Distinguishing CMML from a myelodysplastic neoplasm (MDS) may be difficult when cytopenias, multilineage dysplasia, and increased blasts dominate the initial presentation. In such cases, longitudinal blood counts may be critical for establishing the persistence and diagnostic significance of monocytosis.

Mature plasmacytoid dendritic cell proliferation (MPDCP) associated with a myeloid neoplasm is recognized in the fifth edition of the World Health Organization Classification of Haematolymphoid Tumours (WHO-HAEM5). It occurs most often in association with CMML, although it may also accompany MDS and acute myeloid leukemia (AML) (1, 3–5). Distinction from reactive pDC hyperplasia, blastic plasmacytoid dendritic cell neoplasm (BPDCN), and AML with pDC expansion requires integration of morphology, immunophenotype, clinical distribution, and the features of the underlying myeloid neoplasm because no single immunophenotypic marker is diagnostic (3, 5–7).

The coexistence of increased blasts, dysplasia, monocytosis, and pDC expansion may therefore create two related diagnostic problems, involving classification of the underlying myeloid neoplasm and characterization of the pDC proliferation. We describe an 88-year-old man initially classified as MDS with increased blasts-2 (MDS-IB2) with MPDCP, in whom review of longitudinal hematologic findings supported reclassification as CMML-2 with MPDCP.

## Case presentation

In August 2025, an 88-year-old man with a history of hypertension, atrial fibrillation and chronic constipation was admitted to the Department of Anorectal Surgery after two weeks of worsening constipation and fecal impaction. Circulating blasts detected on admission prompted hematology consultation and transfer for further evaluation. The patient also reported cough and exertional dyspnea. Chest computed tomography showed chronic bronchitis and emphysema, accompanied by findings consistent with pulmonary infection and bilateral pleural effusions. Inflammatory markers were elevated, including C-reactive protein (39.20 mg/L), interleukin-6 (29.08 pg/mL) and procalcitonin (0.12 ng/mL), and piperacillin/tazobactam was administered. Physical examination revealed no skin lesions, lymphadenopathy, or organomegaly. There was no history of blood transfusion and no family history of hematologic malignancy.

Initial laboratory testing showed a white blood cell (WBC) count of 11.67×10^9^/L, AMC of 4.12×10^9^/L (35.3%), hemoglobin (Hb) of 74 g/L, and platelets (PLT) of 79×10^9^/L. Peripheral blood morphology showed approximately 3% blasts, 8% pDC-like cells, and 25% atypical monocytes (**Figures 1A, B**). Bone marrow morphology showed dysplastic hematopoiesis, with myeloblasts accounting for 13.5% of nucleated cells. Additionally, a separate population of pDC-like cells, accounting for approximately 13%, exhibited pale blue cytoplasm, occasional cytoplasmic extensions, and prominent nucleoli (**Figure 1C**). Mature monocytes accounted for 8% of marrow nucleated cells, while no appreciable increase in monoblasts or promonocytes was identified on morphologic differential counting. Peroxidase (POX) cytochemical staining was positive in approximately 26% of myeloblasts but negative in the pDC-like cells (**Figure 1D**). The detailed marrow findings are summarized in **Supplementary Table S1**.

**Figure 1:**
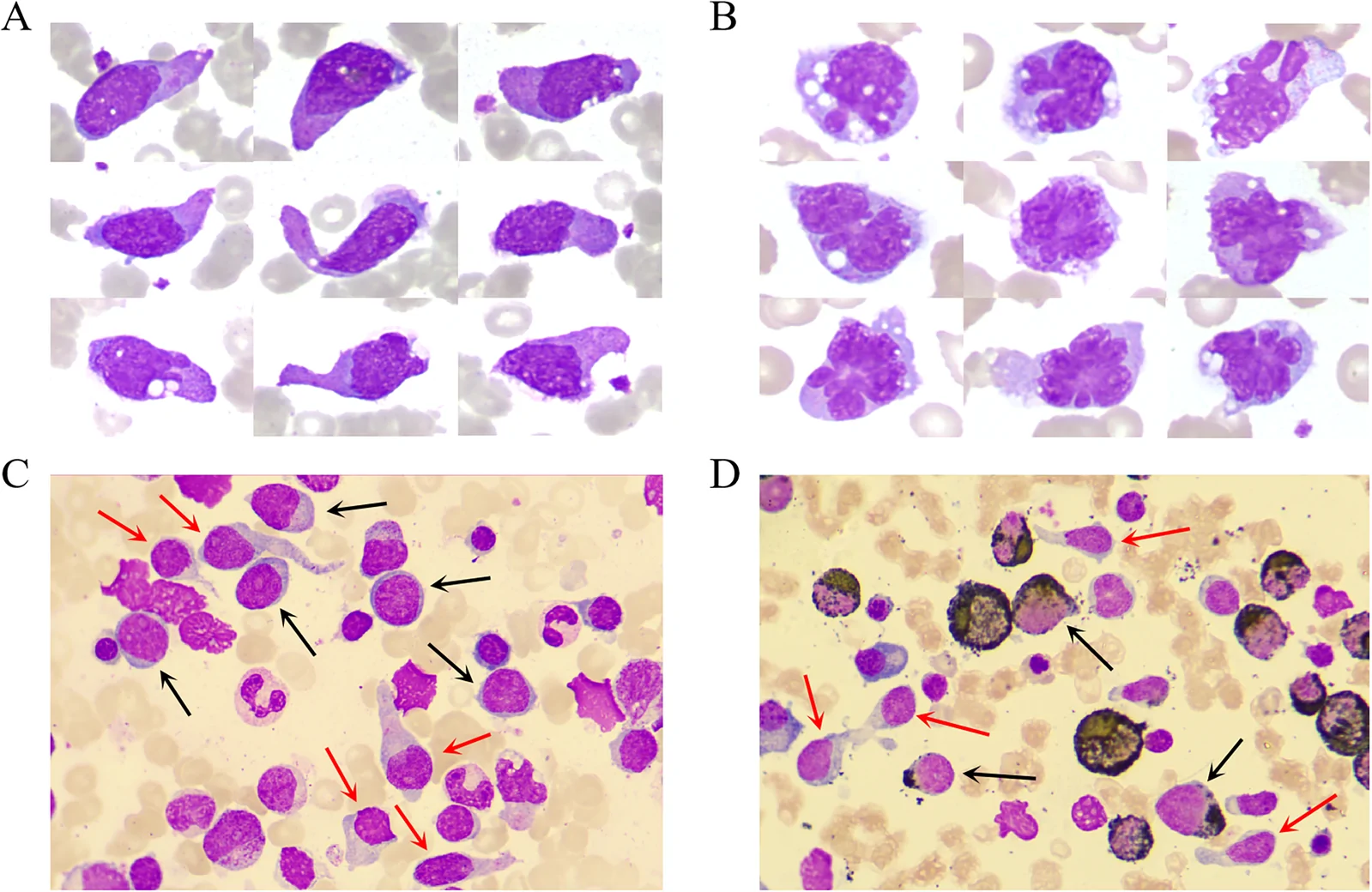
Peripheral blood and bone marrow morphology at initial presentation. **(A)** Representative pDC-like cells and **(B)** atypical monocytes in Wright-Giemsa stained peripheral blood smear. **(C)** Wright-Giemsa and **(D)** peroxidase (POX) cytochemistry staining of bone marrow aspirate smear. Black arrows indicate myeloblasts, and red arrows indicate pDC-like cells. Magnification, ×1,000.

The bone marrow biopsy was hypercellular for the patient’s age, with approximately 40% cellularity. Increased immature-appearing cells were present in clustered and scattered distributions. Megakaryocytic dysplasia was observed, including micromegakaryocytes and hypolobated forms, consistent with the MDS-like megakaryocytic morphology described in CMML (8). CD34 immunohistochemistry highlighted small vessels and rare round nucleated cells, whereas CD117 staining highlighted clustered and scattered positive cells. CD123 staining revealed scattered positive cells, while CD56 showed no appreciable expression (**Supplementary Figure S1**). These findings were supportive but were not used to define the pDC population. Myeloperoxidase (MPO) staining highlighted granulocytic cells, reticulin staining showed focal grade 1 fibrosis (MF-1), and both extracellular and intracellular iron staining were negative (**Supplementary Figure S2**).

Bone marrow flow cytometry identified two phenotypically distinct abnormal populations. An aberrant immature myeloid population accounted for 3.54% of gated cells and expressed CD117 together with the myeloid-associated markers CD13 and CD33. A separate pDC-like population accounted for 8.60% and showed bright CD123 and HLA-DR expression, while lacking CD34 and CD117 (**Figures 2A–F**). The pDC phenotype was further characterized by CD4 and CD303 expression, partial CD304 expression, and absence of CD56 (**Figures 2G–I**). The monocyte population expressed CD64 and CD14, with partial CD16 expression (**Figures 2J–L**).

**Figure 2:**
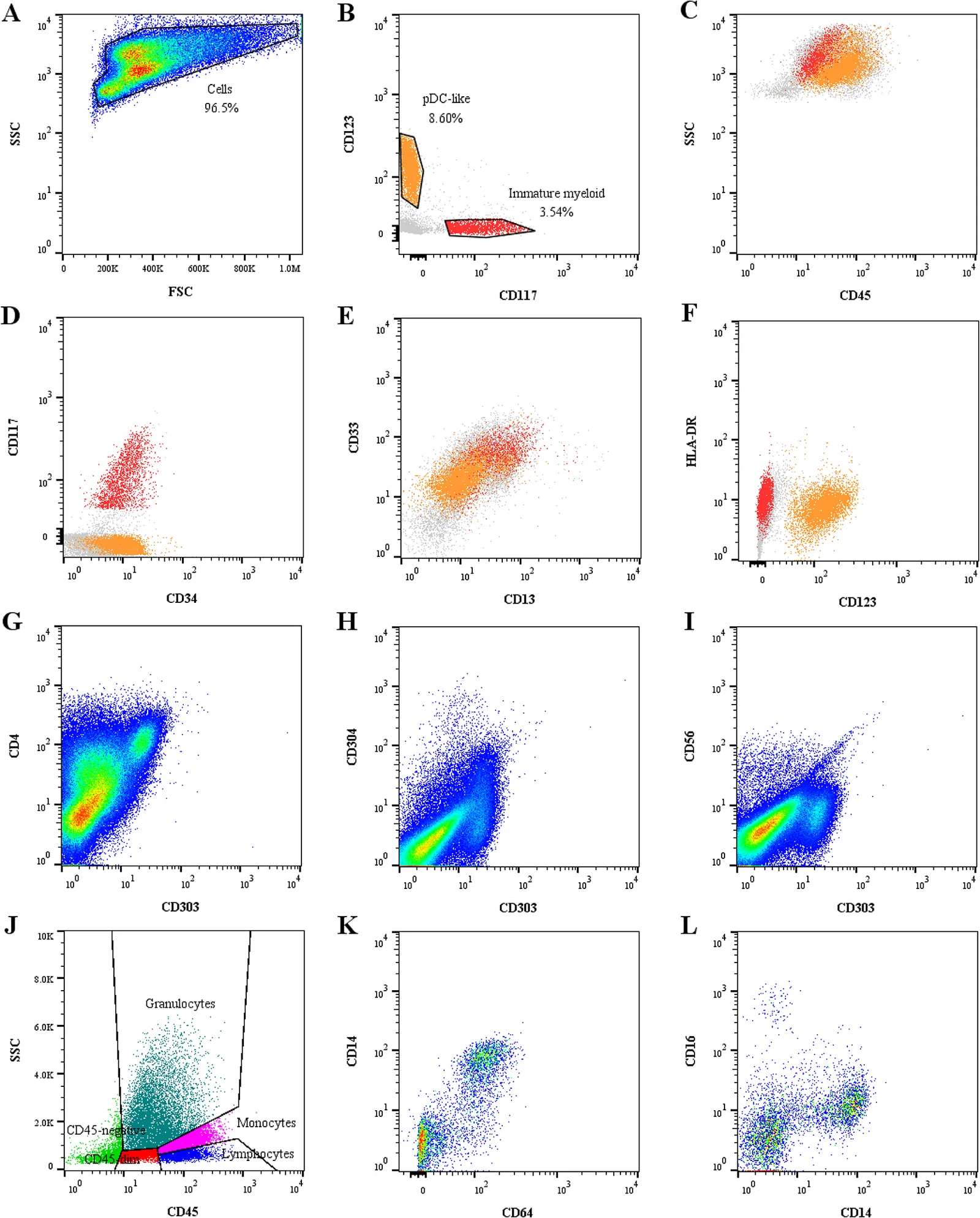
Bone marrow flow-cytometric characterization of the immature myeloid, pDC-like and monocytic populations. **(A)** FSC/SSC gating of the analyzed cell population. **(B)** CD117/CD123 plot identifying a distinct immature myeloid population (3.54%, red) and a pDC-like population (8.60%, orange). **(C)** Distribution of the two populations in CD45/SSC space. **(D–F)** Further characterization of the two populations by CD34/CD117, CD13/CD33 and CD123/HLA-DR expression. **(G–I)** Characterization of the pDC phenotype by CD303/CD4, CD303/CD304, and CD303/CD56 expression. **(J)** CD45/SSC-based distribution of the major leukocyte compartments. **(K, L)** CD64/CD14 and CD14/CD16 expression within the gated monocyte population. The pDC-like and immature myeloid populations are shown in orange and red, respectively, in panels **(B–F)**.

Cytogenetic analysis revealed a complex karyotype with multiple structural and numerical abnormalities, including monosomy 7 (**Supplementary Figure S3**). Targeted next-generation sequencing using a 24-gene myeloid panel identified *TP53* c.404G>A (p.Cys135Tyr) at a variant allele frequency (VAF) of 36.50% and a low-level *ASXL1* c.1762C>T (p.Gln588Ter) nonsense variant at 1.36%. The *ASXL1* variant met the laboratory quality control criteria. Testing for *DEK::NUP214*, *RUNX1::MECOM* and *RUNX1::RPL22* fusions, as well as *BCR::ABL1* transcripts was negative. Copy-number analysis also identified multiple abnormalities, supporting the overall genomic complexity. The complete molecular testing scope and results are summarized in **Supplementary Table S2**.

The patient was initially classified as MDS-IB2 with MPDCP on the basis of marrow dysplasia and 13.5% myeloblasts, a blast percentage within the WHO-HAEM5 MDS-IB2 range, together with a distinct population of morphologically and immunophenotypically mature pDCs. Given his prolonged bed rest and limited daily activity, his Eastern Cooperative Oncology Group (ECOG) performance status was estimated as 3 (9). The patient and his family declined chemotherapy, and management focused on treatment of the pulmonary infection, nutritional support, and symptomatic care. During follow-up, leukocytosis and monocytosis increased substantially. In October 2025, the WBC had risen to 13.21×10^9^/L, with AMC of 5.51×10^9^/L (41.7%). In February 2026, the WBC reached 99.35×10^9^/L, with AMC of 36.76×10^9^/L (37%), Hb of 67 g/L, and PLT of 46×10^9^/L (**Figures 3B–D**). Peripheral blood morphology at this time showed 3% myeloblasts, 1% monoblasts, and 13% promonocytes, corresponding to a combined blast-equivalent proportion of 17% (**Supplementary Figure S4**). Hydroxyurea was administered for cytoreduction, after which the WBC decreased to 20.72×10^9^/L, although the AMC remained elevated at 7.87×10^9^/L (38%). The patient subsequently died during follow-up in April 2026 (**Figure 3A**).

**Figure 3:**
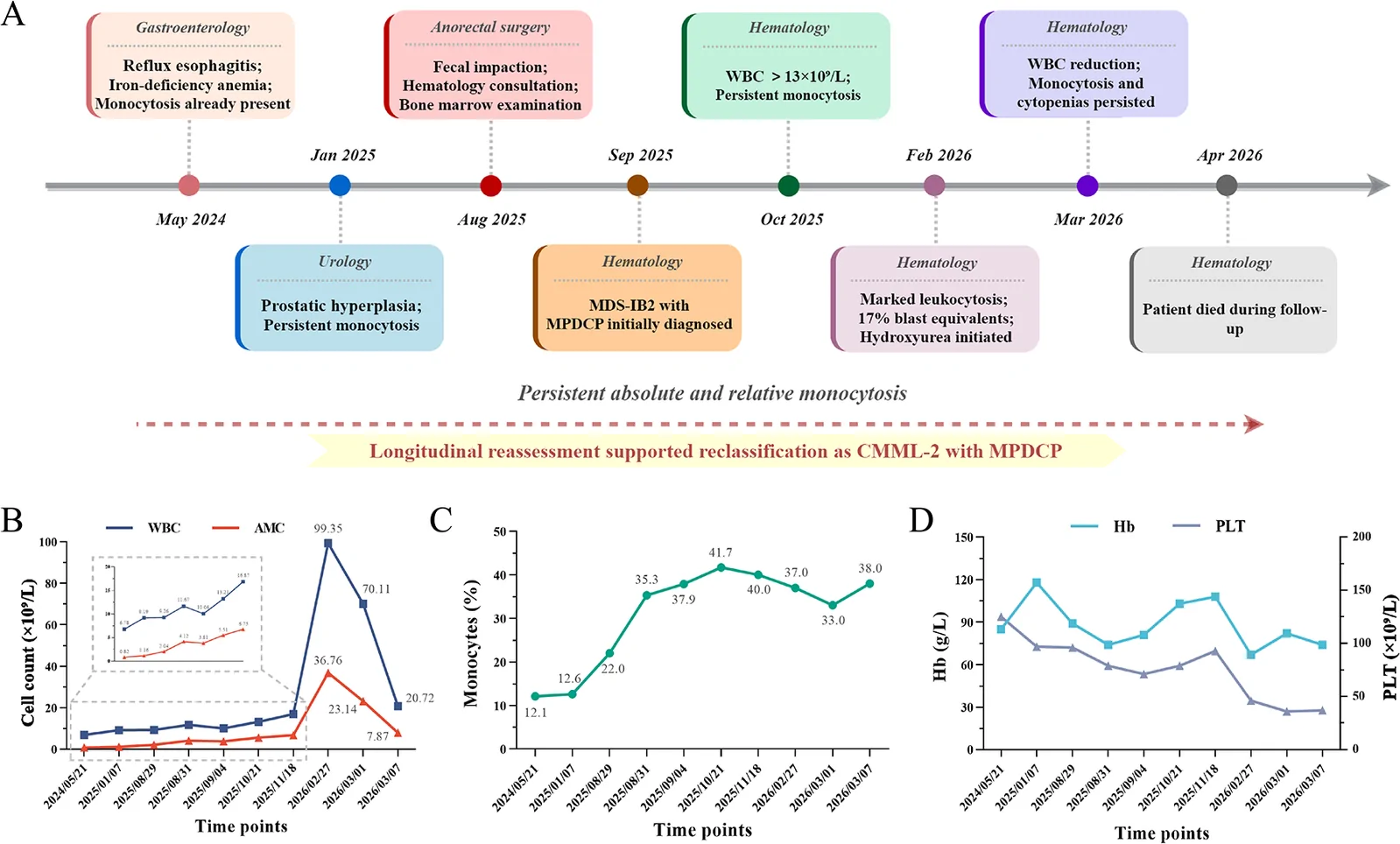
Clinical course and longitudinal hematologic changes. **(A)** Timeline of the major diagnostic, therapeutic, and clinical events. **(B)** Serial white blood cell count (WBC) and absolute monocyte count (AMC). **(C)** Serial relative monocyte percentages. **(D)** Serial hemoglobin (Hb) and platelet (PLT) counts. Detailed hematologic findings are provided in **Supplementary Table S3**.

The findings in February 2026 prompted a retrospective review of the earlier medical records and initial bone marrow examination. Absolute and relative monocytosis had preceded the initial marrow examination, with AMC values of 0.82×10^9^/L (12.1%) in May 2024 and 1.16×10^9^/L (12.6%) in January 2025. The earlier anemia was accompanied by biochemical evidence of iron deficiency (Hb, 85 g/L; serum iron, 1.9 μmol/L; ferritin, 20.70 ng/mL; and a positive fecal occult blood test), and was treated with intravenous iron sucrose. At those earlier encounters, clinical management focused on the documented anemia and the presenting gastrointestinal and urinary disorders, including severe esophagitis and chronic urinary retention. The monocytosis was relatively mild and predominantly involved mature monocytes, without other reported abnormal circulating cells, and CMML was not established. By August 2025, the monocytosis had become more pronounced and was accompanied by worsening cytopenias. The persistent monocytosis, together with the original marrow morphology and evidence of clonality, supported retrospective reclassification as CMML-2 with MPDCP. The complete clinical course and longitudinal hematologic findings are summarized in **Figure 3** and **Supplementary Table S3**.

## Patient perspective

The patient and his family were informed about the diagnosis, prognosis, and available treatment options. They declined chemotherapy and chose supportive care focused on symptom control and quality of life. Hydroxyurea was later accepted when marked leukocytosis required cytoreduction.

## Discussion

The principal diagnostic challenge in this case centered on two related issues involving the classification of the underlying myeloid neoplasm and the nature of the accompanying pDC proliferation. The initial MDS-IB2 diagnosis reflected the prominent dysplastic features and a marrow myeloblast percentage of 13.5%. Population-based studies indicate that MDS is substantially more common than CMML, and this difference in incidence may influence the initial diagnostic probability when clinicopathologic information is incomplete (10, 11). However, retrospective review revealed that monocytosis had been present for more than one year before the marrow examination.

The distinction between MDS and CMML was central to the diagnostic reassessment. Under WHO-HAEM5, persistent absolute monocytosis of at least 0.5×10^9^/L together with relative monocytosis of at least 10% constitutes a prerequisite criterion for CMML. When the AMC is ≥0.5×10^9^/L but <1.0×10^9^/L, both dysplasia and an acquired clonal cytogenetic or molecular abnormality are required as supporting criteria, whereas at an AMC ≥1.0×10^9^/L, at least one supporting criterion is required (1, 8). Abnormal partitioning of peripheral blood monocyte subsets is also recognized by WHO-HAEM5 as a supporting criterion for CMML, although this analysis was not performed in our patient (1, 12). The May 2024 and January 2025 blood counts met both monocytosis thresholds. Because monocytosis had been documented before the August 2025 pulmonary infection, infection may have accentuated the monocytosis at presentation but could not account for the preceding abnormalities. The earlier anemia had been evaluated in the context of iron deficiency, which provided a plausible explanation for part of the hematologic presentation but did not account for the persistent monocytosis. Marrow dysplasia, including MDS-like megakaryocytic changes, increased blasts and clonal genetic abnormalities may occur in both MDS and CMML and therefore do not by themselves distinguish the two entities (8). When interpreted together with the longitudinal monocytosis, the subsequently documented marrow dysplasia and clonal genetic abnormalities supported retrospective classification as CMML-2 rather than a subsequent transformation from MDS to CMML. A recent reclassification study similarly showed that a substantial proportion of patients previously diagnosed with MDS met 2022 CMML criteria after application of the revised monocytosis thresholds, underscoring the diagnostic overlap between these entities (13). A similar diagnostic pitfall was evident in our patient, in whom prominent dysplasia and increased blasts initially drew attention away from the longstanding monocytic phenotype.

The marrow blast count was also important for CMML-2 subclassification. Under WHO-HAEM5 and the ICC, CMML-2 is defined by 5%-19% blasts and blast equivalents in peripheral blood or 10%-19% blasts in bone marrow. In WHO-HAEM5, blast equivalents include myeloblasts, monoblasts and promonocytes (1, 2). The 13.5% marrow myeloblasts in our patient therefore fell within the CMML-2 range. In contrast, flow cytometry detected an aberrant immature myeloid population accounting for 3.54% of gated cells. These measurements were obtained using different methods and should not be considered interchangeable. Blast enumeration is subject to sampling and methodological variation, and discordance between different assessment platforms is well recognized (1, 14). Flow cytometry also delineated a monocyte compartment with a CD64/CD14-positive phenotype and partial CD16 expression. This finding was descriptive and was not used to quantify marrow monocytosis or establish the diagnosis of CMML. CD34 immunohistochemistry likewise cannot replace morphologic blast enumeration, particularly because monoblasts and promonocytes may lack CD34 expression. Although no appreciable increase in these immature monocytic forms was identified in the original marrow examination, their contribution should be considered when assessing the blast-equivalent count in CMML. The pDC-like population was morphologically distinct and should not be incorporated into the myeloid blast count. Accurate separation of these populations is essential to avoid either overestimating or underestimating the proportion of blasts.

Peripheral blood morphology in February 2026 identified 3% myeloblasts, 1% monoblasts, and 13% promonocytes, yielding a combined blast-equivalent proportion of 17% and providing additional morphologic support for CMML-2. This proportion remained below the conventional 20% threshold for AML transformation, although subsequent marrow blast burden could not be assessed because no repeat bone marrow examination was performed. At initial presentation in August 2025, the WBC count was 11.67×10^9^/L, corresponding retrospectively to MD-CMML under the WHO-HAEM5 WBC-defined subtype. By October 2025, it had increased to ≥13×10^9^/L, meeting the threshold for MP-CMML (1, 15). WHO-HAEM5 recognizes these as clinically and genetically distinct CMML phenotypes, with MP-CMML associated with adverse clinical outcomes (1). Thus, the later leukocytosis reflected a clinically relevant shift toward a proliferative phenotype rather than, by itself, evidence of AML transformation (16).

The accompanying pDC proliferation posed a separate diagnostic challenge. Flow cytometry demonstrated a pDC-like population phenotypically distinct from the aberrant immature myeloid population. Across the flow-cytometric panels, the pDC-like phenotype was characterized by bright CD123 and HLA-DR expression, absence of CD34 and CD117, expression of CD4 and CD303, partial CD304 expression, and lack of CD56. Mature pDCs, BPDCN cells, and pDCs associated with acute leukemia may share expression of CD4, CD123, CD303, and CD304, making interpretation of individual markers insufficient (17–19). In this patient, the relatively mature pDC morphology together with this immunophenotypic profile favored mature pDC differentiation. Taken together with the presence of an associated clonal myeloid neoplasm, these findings supported MPDCP, while alternative diagnoses including BPDCN, AML with pDC expansion, and reactive pDC hyperplasia were also considered. The absence of skin lesions and the lack of CD56 expression provided additional context but could not independently exclude BPDCN. CD56-negative BPDCN has been documented, and an extended panel incorporating markers such as TCF4, TCL1, SOX4, and TdT was unavailable in this case (17, 19). Therefore, the distinction rested on the combined morphologic, immunophenotypic, and clinical findings rather than any single negative marker. The differential diagnosis of MPDCP, BPDCN, reactive pDC hyperplasia, and AML with pDC expansion is summarized in **Table 1**.

**Table 1:** Differential diagnosis of pDC proliferations in the present case.

Entity	Morphology	Typical setting	Key immunophenotype	Present case
MPDCP (1, 3, 4, 19)	Mature pDCs	Myeloid neoplasm, especially CMML	CD4^+^, CD123^bright, CD303^+^/CD304^+^; usually CD56⁻, CD34⁻, CD117⁻	Favored: mature morphology, characteristic phenotype and concurrent CMML-2
BPDCN (1, 17–19)	Blastoid cells	Skin, bone marrow, peripheral blood or lymph nodes	CD4^+^, CD123^bright, TCF4^+^, TCL1^+^; CD56 is usually expressed but may be absent	Less supported: mature morphology; no skin lesions; TCF4 and TCL1 not tested
Reactive pDC hyperplasia (4, 17)	Mature pDCs	Infection, inflammation or autoimmune disease	CD4^+^, CD123^bright, CD303^+^/CD304^+^; usually CD56⁻	Not excluded: concurrent infection; pDC clonality unconfirmed
AML with pDC expansion (5, 6)	Myeloid blasts with expanded pDCs	Established AML; often with *RUNX1* mutation	CD4^+^, CD123^bright, CD303/CD304 variable; CD34 variable	Not established: bone marrow myeloblasts below 20%; no AML-defining alteration identified within the available testing

pDC, plasmacytoid dendritic cell; MPDCP, mature plasmacytoid dendritic cell proliferation; BPDCN, blastic plasmacytoid dendritic cell neoplasm; CMML, chronic myelomonocytic leukemia; AML, acute myeloid leukemia.

Reactive pDC expansion also remained a consideration because of the concurrent pulmonary infection. Reactive pDCs may retain a mature immunophenotype and have been reported in infectious and inflammatory conditions (4, 20). The substantial pDC expansion in a marrow showing dysplasia, increased myeloblasts, and an established clonal myeloid neoplasm favored an association with the underlying neoplasm. However, a reactive contribution could not be completely excluded.

Clonal relationships between pDCs and myeloid cells have been demonstrated in some patients with CMML. Lucas et al. demonstrated shared RAS-pathway mutations in sorted pDCs and monocytes from patients with CMML, supporting a common clonal origin (3). Shared *ASXL1* and *CBL* mutations have also been detected in bone marrow and lymph node tissue from a patient with CMML and nodal MPDCP (21). Similar findings in other hematologic neoplasms suggest that mature pDC proliferation may arise from a common hematopoietic clone in some cases (7, 22, 23). These studies are summarized in **Supplementary Table S4**. However, molecular testing in our patient was performed on unsorted marrow, and cytogenetic analysis did not establish which lineage carried monosomy 7. Neither the *TP53* mutation nor monosomy 7 could therefore be assigned specifically to the pDC population. Demonstrating shared clonality would have required sequencing of purified cell populations, lineage-specific fluorescence *in situ* hybridization, or single-cell genomic analysis. Thus, the clinicopathologic findings supported MPDCP, although a shared clonal origin with the myeloid population could not be demonstrated.

Monosomy 7 and the *TP53* mutation supported clonality of the myeloid neoplasm but were not disease-specific. The conventional karyotype and copy-number analysis provided complementary but not directly equivalent information. The copy-number findings supported the overall genomic complexity but did not replace conventional cytogenetic analysis. The *ASXL1* nonsense variant is included in CPSS-Mol and the Mayo Molecular Model, but the prognostic contribution of this low-frequency variant in an individual patient remains uncertain. *TP53* is not included in CPSS-Mol, but its presence alongside a complex karyotype provided additional biologic context when interpreting the patient’s overall risk (24). In addition, add(3)(q21) was not fully characterized. Although testing for *RUNX1::MECOM* was negative, this did not exclude other *MECOM* rearrangements. The genetic findings were therefore interpreted within the scope of the assays performed.

Reclassification from MDS-IB2 to CMML-2 also had implications for clinical assessment and management. The later marked leukocytosis indicated a shift toward a proliferative CMML phenotype. Higher-risk MDS and CMML share some therapeutic approaches, but proliferative CMML may additionally require cytoreduction. In the DACOTA randomized trial, decitabine achieved a higher response rate than hydroxyurea in advanced proliferative CMML but did not improve event-free or overall survival (25). Management was directed primarily at the underlying CMML rather than the pDC proliferation. His poor performance status, corresponding to ECOG 3, was an important consideration in treatment selection. Advanced age, comorbidities, concurrent infection, and severe cytopenias further limited the feasibility of intensive therapy. Hypomethylating therapy was not pursued, and hydroxyurea was used when marked leukocytosis developed. Although it cannot be determined whether earlier recognition of CMML would have altered survival, earlier classification may have facilitated disease-specific risk assessment and interpretation of the subsequent proliferative course.

The prognostic significance of MPDCP itself remains uncertain. Increased clonal pDC populations have been associated with a higher risk of leukemic progression in CMML, but these observations do not establish that pDC expansion independently drives disease progression (3). Published cases vary in their underlying neoplasms, anatomical distribution, molecular findings, and clinical outcome (21, 22, 26–28). Retrospective prognostic assessment at initial presentation classified the patient as high risk across the alternative CPSS (4 points) (29), CPSS-Mol (4 points) (30), and Mayo Molecular Model (4 risk factors) (31) (**Supplementary Table S5**). The concordant high-risk classification across these models indicated a substantial adverse prognostic burden from the underlying CMML and provided context for the subsequent clinical deterioration. The patient’s short survival should therefore be interpreted in the context of this adverse CMML risk profile together with advanced age, comorbidities, concurrent infection, severe cytopenias and limited disease-directed treatment, rather than being attributed solely to the initial MDS-IB2 classification. Neither clonality of the pDC population nor subsequent AML transformation was demonstrated in the present patient. Consequently, the independent prognostic contribution of MPDCP could not be distinguished, and the relative contributions of the underlying CMML and other clinical factors to the patient’s outcome remain uncertain.

Several limitations should be acknowledged. Peripheral blood monocyte subset repartitioning was not performed, an extended BPDCN immunophenotypic panel was unavailable, and the pDC population was not isolated for molecular analysis. Although the later peripheral blood differential provided additional blast-equivalent data, no repeat marrow examination was obtained during the proliferative phase. The molecular workup did not include rearrangement testing for *PDGFRA*, *PDGFRB*, *FGFR1* or *JAK2*. These limitations prevented definitive assessment of pDC clonality, marrow evolution during the later proliferative phase, and complete exclusion of alternative genetically defined myeloid neoplasms.

## Conclusion

When mature pDC expansion accompanies a myeloid neoplasm, both the pDC population and the underlying myeloid disorder should be evaluated in parallel. In this patient, longitudinal monocytosis together with marrow and clonal findings supported reclassification from MDS-IB2 to CMML-2, while morphologic and immunophenotypic findings supported concurrent MPDCP. The case highlights the importance of integrating longitudinal hematologic findings with pDC characterization and of distinguishing clinicopathologic association from molecularly proven clonal identity.

## Data Availability

All de-identified data supporting the findings of this case report are included in the article and its **Supplementary Materials**.
