# Supplementary material for: Case Report: Chronic myelomonocytic leukemia with mature plasmacytoid dendritic cell proliferation initially classified as MDS-IB2
Source: Front Oncol. 2026 Oct 22;16:1961676. doi: 10.3389/fonc.2026.1961676 (PMC13735559; doi:10.3389/fonc.2026.1961676)
Supplement: Supplementary file 1 [file DataSheet1.docx]

**Supplementary Material**

**Supplementary Figure S1. Bone marrow histology and immunohistochemical findings.**

(A, B) Hematoxylin and eosin (H&E) staining showing bone marrow architecture and cellular morphology. Increased immature-appearing cells and dysplastic megakaryocytes were observed. (C) CD34 staining highlighting small vessels and rare round nucleated cells. (D) CD117 staining showing clustered and scattered positive cells. (E) CD123 staining showing positive cells in the bone marrow. (F) CD56 staining showing no appreciable expression. Magnification: ×100 (A) and ×400 (B–F).

**Supplementary Figure S2. Additional bone marrow staining findings.**

(A) Myeloperoxidase (MPO) immunohistochemistry highlighting granulocytic cells in the bone marrow biopsy. (B) Reticulin staining showing focal grade 1 fibrosis (MF-1). (C) Negative extracellular iron staining. (D) Negative intracellular iron staining. Black arrows indicate representative erythroblasts lacking visible iron granules. Magnification: ×400 (A, B), ×100 (C) and ×1,000 (D).

**Supplementary Figure S3. Conventional cytogenetic findings.**

(A) Representative metaphase spread from the bone marrow. (B) Corresponding karyogram showing a complex abnormal karyotype. The complete cytogenetic result was 47,XY,add(3)(q21),add(5)(q11.2),−7,del(9)(q21),del(13)(q12q14),−18,+mar1,+mar2,+mar3[5]/94,
idem×2[2]/46,XY[5].

**Supplementary Figure S4. Peripheral blood morphology in February 2026.**

(A–D) Representative fields from peripheral blood smears showing myeloblasts (black arrows), monoblasts (orange arrows), promonocytes (green arrows), and mature monocytes (red arrows). Differential counting identified 3% myeloblasts, 1% monoblasts, and 13% promonocytes, yielding a combined blast-equivalent percentage of 17%. Magnification: ×1,000.

**Supplementary Table S1. Bone marrow morphology findings at initial evaluation**

| **Category** | **Parameter** | **Result** | **Reference range (%)** |
| --- | --- | --- | --- |
| Granulocytic series | Myeloblasts | 13.5% | 0.31–0.97 |
|  | Promyelocytes | 5% | 1.51–1.63 |
|  | Neutrophilic myelocytes | 7.5% | 4.45–8.53 |
|  | Neutrophil metamyelocytes | 6% | 5.93–9.87 |
|  | Band neutrophils | 3.5% | 20.22–27.22 |
|  | Segmented neutrophils | 8.5% | 6.52–12.36 |
|  |  |  |  |
| Erythroid series | Proerythroblasts | 2.5% | 0.51–1.33 |
|  | Polychromatic erythroblasts | 14% | 5.50–9.32 |
|  | Orthochromatic erythroblasts | 11.5% | 8.39–13.11 |
|  |  |  |  |
| Lymphoid series | Lymphocytes | 5.5% | 15.74–29.82 |
| Monocytic series | Monocytes | 8% | 2.12–3.88 |
| Plasma cells | Plasma cells | 1.5% | 0.29–1.13 |
| Other cells | pDC-like cells | 13% | – |

**Note:**

Reference ranges were obtained from the reporting laboratory. Differential counts are expressed as percentages of bone marrow nucleated cells. No appreciable increase in monoblasts or promonocytes was identified on morphologic differential counting, and separate percentages were therefore not reported. A em dash (–) indicates that no applicable reference range was provided. pDC, plasmacytoid dendritic cell.

**Supplementary Table S2. Molecular testing scope and results in the present case**

| **Assay component** | **Targets assessed** | **Result** |
| --- | --- | --- |
| Gene variation | ASXL1, DNMT3A, ETV6, EZH2, FLT3, GATA2, IDH1, IDH2, JAK2, KIT, KRAS, NPM1, NRAS, PPM1D, RUNX1, SETBP1, SF3B1, SRSF2, STAG2, TET2, TP53, U2AF1, WT1, and ZRSR2 | TP53 c.404G>A (p.Cys135Tyr), VAF 36.50%  ASXL1 c.1762C>T (p.Gln588Ter), VAF 1.36% |
| Fusion gene | DEK::NUP214, RUNX1::MECOM, RUNX1::RPL22, and BCR::ABL1 | Not detected |
| Copy number variation | 3q, 5q, 7/7q, 8, 11q, 12p, 13q, 17p, 19, 20q, and Y | Losses of 3q, 5q, 7q and 13q, with partial gain of 7q |

****Note:****

All molecular analyses were performed on unsorted bone marrow samples using high-throughput sequencing and real-time PCR platforms. The ASXL1 variant was supported by a sequencing depth of 3,171× and was within the validated detection range of the assay, with a validated limit of detection of ≤1% VAF for single-nucleotide variants.

**Supplementary Table S3. Serial hematologic findings and clinical events**

| **Date** | **WBC**  **(×10⁹/L)** | **AMC**  **(×10⁹/L)** | **Monocytes**  **(%)** | **Hb**  **(g/L)** | **PLT**  **(×10⁹/L)** | **hs-CRP**  **(mg/L)** | **Clinical event** |
| --- | --- | --- | --- | --- | --- | --- | --- |
| **May 21, 2024** | 6.78 | 0.82 | 12.1 | 85 | 125 | 27.69 | Gastroenterology admission; Reflux esophagitis; Iron-deficiency anemia |
| **Jan 07, 2025** | 9.19 | 1.16 | 12.6 | 118 | 97 | 24.22 | Urology admission; Prostatic hyperplasia |
| **Aug 29, 2025** | 9.26 | 2.04 | 22.0 | 89 | 96 | 16.02 | Anorectal surgery admission; Fecal impaction; Hematology consultation |
| **Aug 31, 2025** | 11.67 | 4.12 | 35.3 | 74 | 79 | 39.20 | Hematology inpatient evaluation; Bone marrow examination |
| **Sep 04, 2025** | 10.06 | 3.81 | 37.9 | 81 | 71 | 27.32 | MDS-IB2 with MPDCP initially diagnosed |
| **Oct 21, 2025** | 13.21 | 5.51 | 41.7 | 103 | 79 | 13.14 | Persistent monocytosis; WBC≥13×10⁹/L |
| **Nov 18, 2025** | 16.87 | 6.75 | 40.0 | 108 | 93 | 6.42 | Outpatient hematologic follow-up |
| **Feb 27, 2026** | 99.35 | 36.76 | 37.0 | 67 | 46 | 45.31 | Marked leukocytosis; Hydroxyurea initiated |
| **Mar 01, 2026** | 70.11 | 23.14 | 33.0 | 82 | 36 | 24.13 | Declining WBC during hydroxyurea treatment |
| **Mar 07, 2026** | 20.72 | 7.87 | 38.0 | 74 | 37 | 15.14 | Further WBC reduction; Persistent monocytosis and cytopenias |

**Abbreviations:** WBC, white blood cell count; AMC, absolute monocyte count; Hb, hemoglobin; PLT, platelet count; hs-CRP, high-sensitivity C-reactive protein; MPDCP, mature plasmacytoid dendritic cell proliferation; MDS-IB2, myelodysplastic neoplasm with increased blasts-2.

**Supplementary Table S4. Evidence of clonal relationships between pDCs and myeloid neoplasms**

| **Study** | **Disease** | **Method** | **Main finding** | **Relevance to this case** |
| --- | --- | --- | --- | --- |
| Bodmer et al. (2017)^[1]^ | MDS with MPDCP | Molecular comparison of the myeloid neoplasm and pDC proliferation | Shared *PTPN11* mutation | Supports a common clonal origin in MDS-associated MPDCP |
| Lucas et al. (2019)^[2]^ | CMML with pDC expansion | WES of sorted monocytes and pDCs | Shared RAS-pathway mutation | Direct evidence of clonal pDC expansion in CMML |
| Xiao et al. (2021)^[3]^ | AML with pDC expansion | Genomic analysis of sorted cells; PDX model | pDCs clonally related to leukemic blasts | Supports a leukemic origin of pDCs in AML |
| Gong et al. (2022)^[4]^ | AML with mature pDC proliferation | WES and RNA-seq of sorted cells | Shared mutations in pDCs and myeloid blasts | Confirms clonal relationships in mature pDC proliferation |
| Machan et al. (2022)^[5]^ | MDS/CMML with cutaneous pDC proliferation | Molecular comparison of bone marrow and skin lesions | Matching mutations in 2 patients | Supports clonal involvement of extramedullary lesions |
| Al-Nusair et al. (2025)^[6]^ | CMML with nodal MPDCP | NGS of bone marrow and lymph node | Shared *ASXL1* and *CBL* mutations | Tissue-level evidence; pDC-specific clonality not directly tested |

**Note:**

Studies are representative rather than exhaustive. Sequencing of sorted cells provides more direct evidence of pDC clonality than sequencing of unsorted tissue.

**Abbreviations:** pDC, plasmacytoid dendritic cell; MPDCP, mature plasmacytoid dendritic cell proliferation; MDS, myelodysplastic neoplasm; CMML, chronic myelomonocytic leukemia; AML, acute myeloid leukemia; WES, whole-exome sequencing; NGS, next-generation sequencing; PDX, patient-derived xenograft; RNA-seq, RNA sequencing.

**Supplementary Table S5. Retrospective prognostic assessment using three prognostic models**

| **Prognostic variable** | **Scoring criterion** | **Patient's finding** | **Alternative CPSS**^[7]^ | **CPSS-Mol**^[8^**^]^** | **Mayo Molecular Model**^[9]^ |
| --- | --- | --- | --- | --- | --- |
| WHO subtype | CMML-2 | CMML-2 | 1 | — | — |
| WBC count | ≥13×10⁹/L | 11.67×10⁹/L | 0 | 0 | — |
| Bone marrow blasts | ≥5% | 13.5% | — | 1 | — |
| Cytogenetic risk | High | Complex karyotype; −7 | 2 | 2^*^ | — |
| Truncating *ASXL1* mutation | Present | p.Gln588Ter; VAF 1.36% | — | 1^*^ | 1 |
| Genetic risk group | Genetic score ≥3 | High; genetic score of 3 | — | 3 | — |
| RBC transfusion dependence | Present | No | — | 0 | — |
| Hemoglobin | <10 g/dL | 7.4 g/dL | 1 | — | 1 |
| Platelet count | <100×10⁹/L | 79 × 10⁹/L | — | — | 1 |
| Absolute monocyte count | >10×10⁹/L | 4.12 × 10⁹/L | — | — | 0 |
| Circulating immature myeloid cells | Present | 3% blasts | — | — | 1 |
| Total score |  |  | 4 | 4 | 4 |
| Risk category |  |  | High | High | High |

**Notes:**

(a) High-risk categories were defined as ≥4 points for the alternative CPSS and CPSS-Mol, and ≥3 risk factors for the Mayo Molecular Model.
(b) Cytogenetic risk was classified as high because of monosomy 7 and a complex karyotype.
(c) The 24-gene NGS panel detected an *ASXL1* nonsense variant (p.Gln588Ter; VAF, 1.36%) that met laboratory quality-control criteria. No reportable mutations were detected in *NRAS*, *RUNX1*, or *SETBP1*.
(d) ^*^ In CPSS-Mol, cytogenetic and molecular findings were combined into the genetic score and were not counted separately.
(e) the genetic score of 3 corresponded to a high genetic risk group, contributing 3 points to the final score. Individual genetic points were not counted twice.

**Abbreviations:** AMC, absolute monocyte count; CMML, chronic myelomonocytic leukemia; CPSS, CMML-specific Prognostic Scoring System; CPSS-Mol, molecular CPSS; VAF, variant allele frequency; RBC, red blood cell; WBC, white blood cell count.

**References**

[1]	BODMER A, MENTER T, JUSKEVICIUS D, et al. Sharing of a PTPN11 mutation by myelodysplastic bone marrow and a mature plasmacytoid dendritic cell proliferation provides evidence for their common myelomonocytic origin [J]. Virchows Arch, 2017, 470(4): 469-473. DOI:10.1007/s00428-017-2075-5.

[2]	LUCAS N, DUCHMANN M, RAMEAU P, et al. Biology and prognostic impact of clonal plasmacytoid dendritic cells in chronic myelomonocytic leukemia [J]. Leukemia, 2019, 33(10): 2466-2480. DOI:10.1038/s41375-019-0447-3.

[3]	XIAO W, CHAN A, WAARTS M R, et al. Plasmacytoid dendritic cell expansion defines a distinct subset of RUNX1-mutated acute myeloid leukemia [J]. Blood, 2021, 137(10): 1377-1391. DOI:10.1182/blood.2020007897.

[4]	GONG X, LI C, WANG Y, et al. Mature plasmacytoid dendritic cells associated with acute myeloid leukemia show similar genetic mutations and expression profiles to leukemia cells [J]. Blood Sci, 2022, 4(1): 38-43. DOI:10.1097/bs9.0000000000000097.

[5]	MACHAN S, ALONSO-DOMINGUEZ J M, SáNCHEZ GARCíA F J, et al. Plasmacytoid Dendritic Cell Dermatosis Associated to Myeloproliferative/Myelodysplastic Neoplasms [J]. Am J Surg Pathol, 2022, 46(12): 1623-1632. DOI:10.1097/pas.0000000000001960.

[6]	AL-NUSAIR J, PORTER N, ALAGHA Z, et al. Nodal Mature Plasmacytoid Dendritic Cell Proliferation in a Patient With Chronic Myelomonocytic Leukemia: A Diagnostic Mimic of Blastic Plasmacytoid Dendritic Cell Neoplasm [J]. J Investig Med High Impact Case Rep, 2025, 13: 23247096251344723. DOI:10.1177/23247096251344723.

[7]	SUCH E, GERMING U, MALCOVATI L, et al. Development and validation of a prognostic scoring system for patients with chronic myelomonocytic leukemia [J]. Blood, 2013, 121(15): 3005-3015. DOI:10.1182/blood-2012-08-452938.

[8]	ELENA C, GALLì A, SUCH E, et al. Integrating clinical features and genetic lesions in the risk assessment of patients with chronic myelomonocytic leukemia [J]. Blood, 2016, 128(10): 1408-1417. DOI:10.1182/blood-2016-05-714030.

[9]	PATNAIK M M, ITZYKSON R, LASHO T L, et al. ASXL1 and SETBP1 mutations and their prognostic contribution in chronic myelomonocytic leukemia: a two-center study of 466 patients [J]. Leukemia, 2014, 28(11): 2206-2212. DOI:10.1038/leu.2014.125.
